# Mechanical Dentistry

**Published:** 1877-07

**Authors:** C. B. Porter


### MECHANICAL DENTISTRY.

BY DR. C. B. PORTER.

A few thoughts came into my mind after reading the discussions on Mechanical Dentistry, "Celluloid, its advantages, etc," by the Ohio State Dental Society. Now, I feel an old time interest in this department of dental practice. There are many dentists, no doubt, who dislike the idea of overlooking so important a part of our professional duties. To bring up in our dental societies a topic for discussion and call it mechanical dentistry, and pass it off with a few remarks, on which is the best of two of the cheapest articles we have to use for the base plate for artificial teeth, and dismiss the subject as unworthy of further notice, not thinking they are treading on the verge of truths as deep and important as any in the whole range of dental science. To be a skillful mechanical dentist, one should have a clear knowledge of the characteristics of the teeth, their appearance in the mouth, also their marked and striking differences. It is in the operative department where we have the best and almost only advantages of studying the natural teeth closely in the mouths of persons of different temperaments. observing their characteristics, etc., for future use. A mechanical dentist should be an anatomist, a physiologist, as well general as special, and the more he knows of the general principals of physiognomy the better. Every one should know that the teeth change in color, length, and arrangement from infancy to old age; also, that there are several well marked classes of teeth, each differing, in some respects, from all others; also many intermediate classes. An excellent plan to indelibly impress this upon the mind, is to note down, from time to time, as interesting cases present themselves to our notice, which can be made useful at any time when persons of similar temperament and physiological developements present themselves for artificial substitutes, remembering the old maxim, "That every part of a thing corresponds with every other part, and with the whole."

Mr. J. T.—aged 19, height 5 feet 5¾ in.; temperament, *bilious*,—color of hair, *black*; color of eyes, *brown*; color of teeth, *dark*; color of skin, slightly *dark*. Length of central incisor teeth to gum, 5 lines. Width of central incisor teeth, 4 lines. Teeth quite prominent, a marked feature of the face. Shape of face inclined to the pyriform. Occupation clerk.

Mr. L. W. aged 72 years; height, 5 feet 11½ in; temperament, nervo-sanguine; color of hair (originally) brown; color of eyes, light gray. Incisor (central) teeth originally light, colored somewhat by age. Width of central incisors, 4 lines. Length to gum, 5 lines. Upper teeth quite prominent and shut well over the under ones. Shape of face, oval. (I removed his remaining teeth for a new set.)

These two examples are rather extreme ones. Almost every individual possesses some marked feature differing from all others, which will require our notice in selecting artificial teeth. In the operative department we will do all we can to save the natural teeth, but if we ignore the fact, it is nevertheless true, that very many people will loose their natural teeth, and substitutes must be made. Would it not be well if dentists would pay the same attention, or more, to mechanical dentistry they did twenty-five years ago, or bring it up on a par with the operative department.

Dr. C. R. Taft, to my mind, struck the right note in his address on leaving the president's chair of the Ohio State Dental Society, when he asks: "Does the mechanical branch stand where we would like to see it?" etc.

So far, I have said but little about the base plate, taking it for granted that every dentist of any intelligence knows that gold for all partial plates, and gold or platinum, (continuous gum work) for full plates are absolutely the best and most durable materials that can be used. Still rubber, celluloid, and cast metal plates have an important place to serve as cheaper articles for those who cannot afford the more costly, gold or continuous gum work. Let us, for humanity's sake, talk more in our societies, when we have mechanical dentistry up for discussion, how to select artificial teeth of the right size, shape, color, and the position they should occupy in the mouth of the patient, use what we may for the plate, that we may no more see those deformities so often met with at every turn. We cannot mingle in any gathering without seeing very unnatural artificial teeth in the mouths of many people. Old ladies and gentlemen with artificial teeth too white, too small, too young, and illy set, a rubber plate with material enough in it for two plates.

I sometimes wish every dentist could be compelled to spend a few months at the great art school of Kensington, England, to see if it were not possible to acquire a taste for the artistic, the true, and the beautiful. Then we might hope to see better work from the dental laboratory, and "Mechanical [artistic] dentistry stand where we would like to see it."

